# The Polymorphism of *YWHAE*, a Gene Encoding 14-3-3Epsilon, and Brain Morphology in Schizophrenia: A Voxel-Based Morphometric Study

**DOI:** 10.1371/journal.pone.0103571

**Published:** 2014-08-08

**Authors:** Mikio Kido, Yukako Nakamura, Kiyotaka Nemoto, Tsutomu Takahashi, Branko Aleksic, Atsushi Furuichi, Yumiko Nakamura, Masashi Ikeda, Kyo Noguchi, Kozo Kaibuchi, Nakao Iwata, Norio Ozaki, Michio Suzuki

**Affiliations:** 1 Department of Neuropsychiatry, University of Toyama, Toyama, Japan; 2 Department of Psychiatry, Nagoya University Graduate School of Medicine, Nagoya, Japan; 3 Department of Neuropsychiatry, Division of Clinical Medicine, Faculty of Medicine, University of Tsukuba, Ibaraki, Japan; 4 Department of Psychiatry, Fujita Health University School of Medicine, Toyoake, Japan; 5 Department of Radiology, University of Toyama, Toyama, Japan; 6 Department of Cell Pharmacology, Nagoya University Graduate School of Medicine, Nagoya, Japan; 7 Core Research for Evolutional Science and Technology, Japan Science and Technology Corporation, Tokyo, Japan; United Graduate School of Child Development, Osaka University, Japan

## Abstract

**Background:**

*YWHAE* is a possible susceptibility gene for schizophrenia that encodes 14-3-3epsilon, a Disrupted-in-Schizophrenia 1 (*DISC1*)-interacting molecule, but the effect of variation in its genotype on brain morphology remains largely unknown.

**Methods:**

In this voxel-based morphometric magnetic resonance imaging study, we conducted whole-brain analyses regarding the effects of *YWHAE* single-nucleotide polymorphisms (SNPs) (*rs28365859, rs11655548, and rs9393*) and *DISC1* SNP (*rs821616*) on gray matter volume in a Japanese sample of 72 schizophrenia patients and 86 healthy controls. On the basis of a previous animal study, we also examined the effect of *rs28365859* genotype specifically on hippocampal volume.

**Results:**

Whole-brain analyses showed no significant genotype effect of these SNPs on gray matter volume in all subjects, but we found significant genotype-by-diagnosis interaction for *rs28365859* in the left insula and right putamen. The protective C allele carriers of *rs28365859* had a significantly larger left insula than the G homozygotes only for schizophrenia patients, while the controls with G allele homozygosity had a significantly larger right putamen than the C allele carriers. The C allele carriers had a larger right hippocampus than the G allele homozygotes in schizophrenia patients, but not in healthy controls. No significant interaction was found between *rs28365859* and *DISC1* SNP on gray matter volume.

**Conclusions:**

These different effects of the *YWHAE* (*rs28365859*) genotype on brain morphology in schizophrenia and healthy controls suggest that variation in its genotype might be, at least partly, related to the abnormal neurodevelopment, including in the limbic regions, reported in schizophrenia. Our results also suggest its specific role among *YWHAE* SNPs in the pathophysiology of schizophrenia.

## Introduction

Schizophrenia is a heterogeneous psychiatric disorder with a multifactorial etiology in which multiple susceptibility genes interact with environmental factors [1], [2]. Convergent evidence from neuroimaging studies in schizophrenia suggests subtle but widespread gray matter (GM) reductions predominantly in the frontal and temporo–limbic regions (e.g., hippocampus), at least partly as a consequence of early neurodevelopmental insult [3], [4]. These brain morphologic changes in schizophrenia could be useful endophenotypes for unraveling the molecular etiopathology of this complex psychiatric disorder [5], [6].

The Disrupted-in-Schizophrenia 1 (*DISC1*) gene [7], [8], which is thought to be involved in mechanisms of neurodevelopment and synaptic plasticity in cortical and limbic regions [9]–[13], has been one of the candidate genes for schizophrenia [14], [15]. In addition to the possible effect of *DISC1* genotype variation on brain function and structure in the hippocampus [16] and cingulate cortex [17] in healthy subjects, our preliminary magnetic resonance imaging (MRI) study suggested that it might differentially affect GM volume of the neocortical and limbic regions in schizophrenia patients and healthy controls [18]. Several other MRI studies of *DISC1* in schizophrenia have yielded inconsistent results [reviewed by Duff et al. [19] and there have also been questions about *DISC1* as a genetic risk factor of schizophrenia [20]. However, *DISC1* interacts with a complex formed by related molecules [13] and the genetic variation in such *DISC1*-interacting molecules might have a significant role in the pathophysiology of schizophrenia.

*YWHAE* is a gene encoding 14-3-3epsilon, one of the *DISC1*-interacting molecules that is thought to play a crucial role in neuronal development via transport of the NudE-like (*NUDEL*)/lissencephaly-1 (*LIS1*) complex [13], [21], and is a possible susceptibility gene for schizophrenia as identified in a Japanese population [22]. Genetic and expression evidence indicated that a functional single-nucleotide polymorphism (SNP) in the 5′ flanking region (*rs28365859*) was associated with schizophrenia, with subjects with the C allele having a reduced risk of the illness [22]. In addition, animal studies using genetically modified 14-3-3epsilon-deficient mice showed developmental defects of hippocampal neurons [21] as well as working memory deficits [22], which is one of the prominent features of schizophrenia [23]. Despite these observations supporting the significant role of *YWHAE* in the neurobiology of schizophrenia, the possible association between variation in its genotype and brain morphology in schizophrenia remains largely unknown.

In this MRI study, we used voxel-based morphometry (VBM), which allows automated whole-brain analysis, to explore the effects of a *YWHAE* SNP (*rs28365859*) on regional GM volume in a Japanese sample of schizophrenia patients and matched healthy controls. On the basis of the potential role of *YWHAE* in neuronal development as well as previous MRI findings in schizophrenia [3], [4], we predicted significant diagnosis-by-genotype interaction predominantly in frontal and temporo–limbic regions, with patients with the protective C allele having a larger GM volume. As previous animal studies suggested the impact of *YWHAE* on the hippocampus [21], we also examined the effect of its genotype specifically on hippocampal volume using small volume correction (SVC) of VBM analyses, with the hypothesis that subjects with the C allele would have a larger hippocampal volume, especially in schizophrenia patients.

To investigate the specificity of the effect of *rs28365859* on brain morphology, we also examined two putative non-risk SNPs in *YWHAE* (*rs11655548* that was associated with schizophrenia but located in the intron region and *rs9393*, a functional SNP with no difference in genotype distribution between schizophrenia and controls) [22]. Possible interaction effect between *rs28365859* and *DISC1* Ser704Cys SNP (*rs821616*) on brain morphology was also examined.

## Methods

### Ethics statement

This protocol was approved by Committee on Medical Ethics of Toyama University and Nagoya University Graduate School of Medicine. After a complete and detail description of the study was given, subjects provided written informed consent. Clinical staff explained the nature of the study to the subjects, the risks and benefits, and the option not to participate in this research. If the mental status of a subject was impaired to the point where s/he could not understand these issues, the subject was not asked to participate in this research. If there was a possibility that the capacity of a participant to consent was compromised, an additional consent form was obtained from the next of kin, care takers, or guardians of such subjects.

### Subjects

Seventy-two patients with schizophrenia (39 males and 33 females; mean age = 27.5 years, SD = 6.0) who met the ICD-10 research criteria [24] were recruited from inpatient and outpatient clinics of the Department of Neuropsychiatry of Toyama University Hospital. The patients were diagnosed following a structured clinical interview by psychiatrists using the Comprehensive Assessment of Symptoms and History (CASH) [25]. Clinical symptoms were rated at the time of scanning using the Scale for the Assessment of Negative Symptoms (SANS) [26] and the Scale for the Assessment of Positive Symptoms (SAPS) [27]. Sixty-eight patients were right-handed and four patients were mixed-handed.

The control subjects consisted of 86 right-handed healthy volunteers (45 males and 41 females; mean age = 26.4 years, SD = 6.6) recruited from members of the local community, hospital staff, and university students. They were asked to complete a questionnaire consisting of 15 items concerning their personal (13 items; including a history of obstetric complications, substantial head injury, seizures, neurological or psychiatric disease, impaired thyroid function, hypertension, diabetes, and substance abuse) and family (2 items) histories of illness. Subjects with any personal or family history of psychiatric illness among their first-degree relatives were excluded.

All subjects were Japanese and physically healthy at the time of the study. None had a lifetime history of serious head trauma, neurological illness, serious medical or surgical illness, or substance abuse. All participants were also screened for gross brain abnormalities by neuroradiologists. The subject overlap with our previous publication included 30/72 schizophrenia patients and 28/86 controls, where we reported the effect of *DISC1* Ser704Cys polymorphism (*rs821616*) on brain morphology [18].

### SNP genotyping

Genomic DNA was extracted from EDTA-containing venous blood samples according to standard procedures. The genotyping of SNPs in *YWHAE* (*rs28365859, rs11655548,* and *rs9393*) and *DISC1* (*rs821616*) was performed by TaqMan assays (Applied Biosystems, Foster City, CA). TaqMan SNP Genotyping Assay and Universal PCR Master Mix were obtained from Applied Biosystems. Allelic-specific fluorescence was measured using the ABI PRISM 7900 Sequence Detector System (Applied Biosystems).

### MRI procedures

MR images were obtained using 1.5 T Magnetom Vision (Siemens Medical System, Inc., Erlangen, Germany) with a three-dimensional gradient-echo sequence FLASH (fast low-angle shots) yielding 160–180 contiguous T1-weighted slices of 1.0 mm thickness in the sagittal plane. The imaging parameters were as follows: repetition time = 24 ms; echo time = 5 ms; flip angle = 40°; field of view = 256 mm; and matrix size = 256×256 pixels. The voxel size was 1.0×1.0×1.0 mm. The scanner was calibrated weekly with the same phantom to ensure measurement stability.

T1-weighted MR images were processed using Statistical Parametric Mapping 8 (SPM8, Wellcome Institute of Neurology, University College London, UK, http://www.fil.icon.ucl.ac.uk/spm) running under MATLAB R2012b (The MathWorks Inc., USA). The images were preprocessed using the VBM8 toolbox (http://dbm.neuro.uni-jena.de/vbm/), which is an extension of the unified segmentation model consisting of spatial normalization, bias field correction, and tissue segmentation [28]. Registration to the stereotactic space of the Montreal Neurological Institute (MNI) consisted of linear affine transformation and nonlinear deformation using high-dimensional Diffeomorphic Anatomical Registration through Exponential Lie Algebra (DARTEL) normalization [29]. Estimation options were set as follows: extremely light bias regulation; bias cut-off full width at half maximum (FWHM) = 30 mm; affine regulation = International Consortium for Brain Mapping (ICBM) space template of East Asian brains; and the others were defaults. The normalized and segmented images were modulated by applying a nonlinear deformation, which allows comparison of absolute amounts of tissue corrected for individual differences in brain size. The bias-corrected, modulated, and warped tissue maps were then written with an isotopic voxel resolution of 1.5×1.5×1.5 mm and smoothed with an 8-mm FWHM Gaussian kernel [30], [31].

### Exploratory whole-brain analysis of regional GM volume

First, we performed whole-brain analyses using SPM8 to explore the effects of genotype and genotype-by-diagnosis interaction for each of *YWHAE* (*rs28365859, rs11655548*, and *rs9393*) and *DISC1* (*rs821616*) SNPs on GM volume in all subjects. These effects were statistically assessed using a full factorial model for a 2×2 ANOVA, with diagnosis and genotype status as independent variables, and age and sex as covariates of no interest in SPM8. In order to avoid type I error, the significance level was set at *p*<0.0001 (uncorrected for multiple comparison), and the extent threshold of cluster size was set at *k*>50. We also explored the gene-gene interaction between *rs28365859* and *rs821616* on brain morphology using a full factorial model for a 2×2 ANOVA, with genotype status of each SNP as independent variables.

Using the Wake Forest University (WFU) PickAtlas [32], we then performed small volume corrections (SVCs) for each brain region including the clusters with a significant genotype effect or interaction. Each region was defined using the Automated Anatomical Labeling (AAL) atlas [33]. For the regions of interest (ROIs) with significant genotype-by-diagnosis interaction, the genotype effect was examined separately in the patients and controls, with age and sex as covariates of no interest. For these SVC analyses, a family-wise error-corrected (FWE) voxel level threshold of *p*<0.05 was applied to account for multiple comparisons of the results. Voxel coordinates were given as an indication of location in a standardized brain. Voxels were localized in MNI space and transformed into Talairach and Tournoux coordinates [34] using the WFU PickAtlas [35], [36].

### Hypothesis-driven ROI analysis for hippocampus

On the basis of a previous postmortem rat experiment [21], we also examined the effect of *rs28365859* on bilateral hippocampi defined by the AAL atlas (FWE, *p*<0.05). For this hypothesis-driven ROI analysis, we examined the effect of genotype in all subjects as well as in each diagnostic group. Age and sex were used as covariates of no interest in these analyses.

### Statistical analysis

Demographic and clinical differences between groups were examined by using chi-square test or one-way analysis of variance (ANOVA) with post hoc Scheffé’s test. Genotypes were tested for Hardy–Weinberg equilibrium (HWE) using the chi-square goodness-of-fit test. Since the number of subjects with C allele homozygosity of *rs28365859* was quite small (3 schizophrenia patients and 4 control subjects), and on the basis of a previous report on lymphocytes of healthy control subjects [22], the study participants were categorized into C allele carriers (protective allele group) or G allele homozygotes. For other *YWHAE* and *DISC1* SNPs, on the basis of minor allele frequency [22] and previous report [18], the subjects were divided into G allele carriers *vs* A allele homozygotes (*rs11655548* and *rs9393*) and T allele homozygotes *vs* A allele carriers (*rs821616*), respectively. Statistical significance was defined as *p*<0.05.

## Results

### Sample characteristics and genotyping results

Groups were matched for age, sex, height, body weight, and total GM volume, but the controls had attained a higher level of education than the schizophrenia patients (Table 1). In Table1, the different typical and atypical antipsychotic dosages were converted into haloperidol equivalent according to the guidelines by Toru [37]. There was no significant difference in clinical and demographic data between *YWHAE (rs28365859)* C allele carriers and G allele homozygotes in both schizophrenia and control groups. The genotype frequencies of the SNPs investigated in this study were within the distribution expected according to the HWE. As shown in Table 1, patients with schizophrenia and healthy comparisons did not differ significantly in genotype distributions (chi-square = 1.62, *p* = 0.204) and allele frequencies (chi-square = 1.00, *p* = 0.317) of *rs28365859*.

**Table 1:** Clinical and *YWHAE* genotypic description of schizophrenia patients and healthy controls.

	Schizophrenia patients		Controls		Group comparisons
	C allele carriers	G homozygotes	C allele carriers	G homozygotes	
	(*n* = 34)	(*n* = 38)	(*n* = 32)	(*n* = 54)	
Male/female	14/20	25/13	19/13	26/28	Chi-square = 3.95, *p* = 0.27
Age (years)	27.2±5.9	27.9±6.2	25.5±6.6	27.0±6.6	*F* (3,154) = 0.85, *p* = 0.47
Height (cm)	162.3±8.7	166.4±8.1	166.9±9.6	164.5±7.4	*F* (3,154) = 2.22, *p* = 0.09
Body weight (kg)	56.3±9.5	62.1±11.6	57.9±9.9	57.1±9.7	*F* (3,154) = 2.48, *p* = 0.06
Education (years)	13.9±1.7	13.6±2.1	16.0±2.2	15.9±2.3	*F* (3,153 ) = 13.79, *p*<0.01;Con>Sz
Parental education (years)	13.0±1.8	12.4±2.5	13.2±2.5	13.3±2.4	*F* (3,153) = 1.22, *p* = 0.30
Age of onset (years)	21.7±4.1	23.3±5.1	-	-	*F* (1,70) = 2.21, *p* = 0.14
Duration of illness (years)	5.4±5.8	4.4±4.6	-	-	*F* (1,70) = 0.64, *p* = 0.43
Duration of medication (years)	2.9±3.9	3.2±3.7	-	-	*F* (1,70) = 0.11, *p* = 0.75
Drug dose (haloperidolequivalent, mg/day)	8.2±7.2	9.3±8.3	-	-	*F* (1,70) = 0.37, *p* = 0.55
Total SAPS scorea)	32.3±26.3	28.3±26.6	-	-	*F* (1,69) = 0.40, *p* = 0.53
Total SANS scorea)	53.1±24.1	52.2±20.6	-	-	*F* (1,69) = 0.03, *p* = 0.87
Total gray matter volume (mm^3^)	631.3±46.6	658.0±64.4	655.6±52.3	654.5±57.2	*F* (3,154) = 1.74, *p* = 0.16

Values represent means ± SDs. Con, controls; SANS, Scale for the Assessment of Negative Sympoms; SAPS, Scale for the Assessment of Positive Symptoms; Sz, schizophrenia.

a) Data missing for one patient.

For the other SNPs, *rs11655548* (3 patients and 3 controls), *rs9393* (3 patients and 1 control), and *rs821616* (3 patients) were not detected for some participants. There was a group difference in the genotype distribution only for *rs9393* (chi-square = 5.65, *p* = 0.018; less G allele carriers in the patients), but such a difference was not found in a larger sample including the current sample (*n* = 332) or in a large independent Japanese sample (*n* = 3157) [22].

### Exploratory whole-brain analysis of regional GM volume

**Figure 1:**
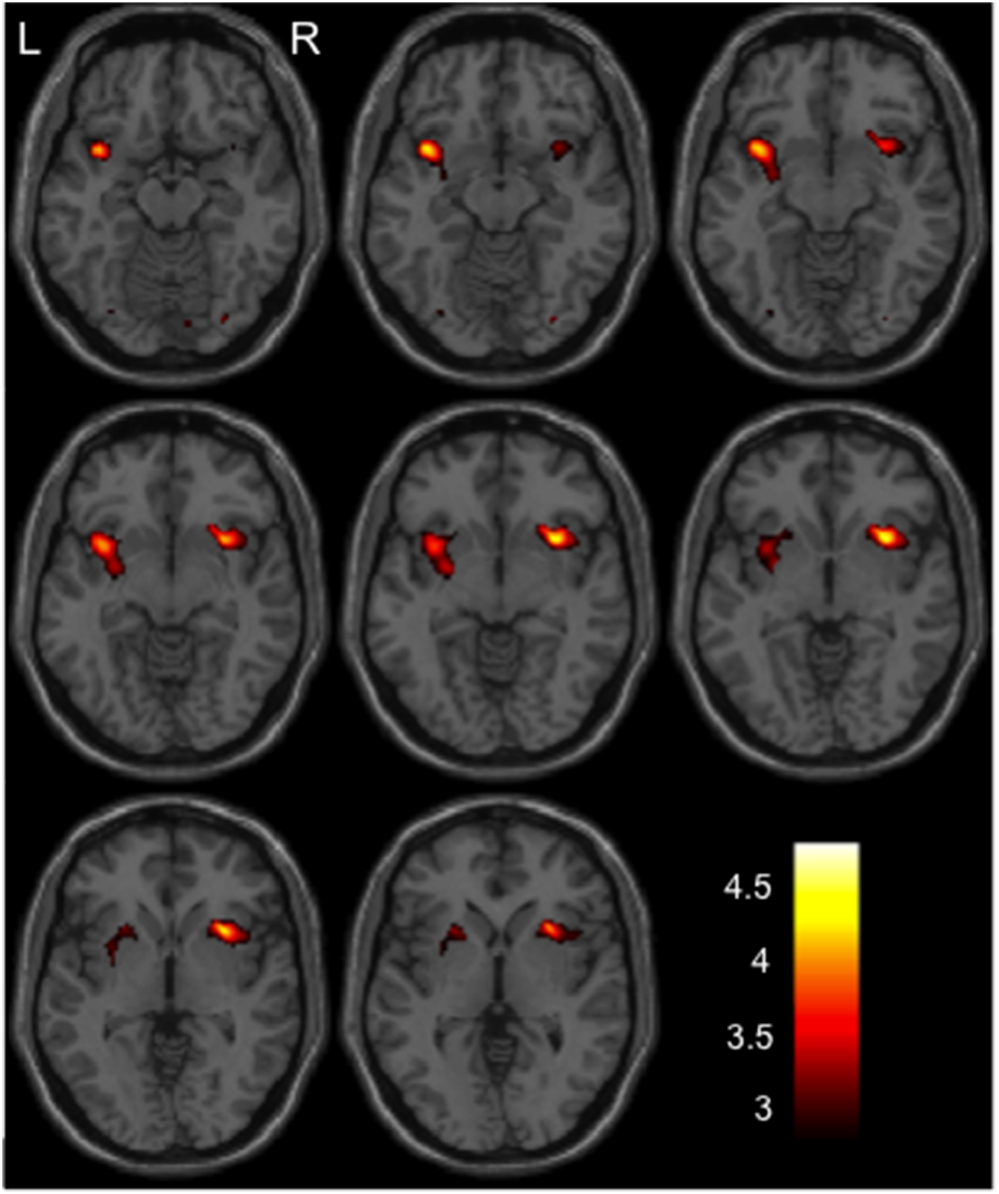
The *YWHAE* (*rs28365859*) genotype-by-diagnosis interaction on gray matter volume. The regions showing interaction in all subjects are displayed by a hot colormap. The color bar shows t values corresponding to the color in the figure.

There was no significant genotype effect of *YWHAE* SNPs or *rs821616* on GM volume in all subjects. However, we found significant genotype-by-diagnosis interactions for *rs28365859* in the left insula and right putamen GM volume (uncorrected *p*<0.0001, extent threshold k>50; Table 2 and Fig. 1), which were confirmed by subsequent FWE-corrected SVC analyses (left insula, *p* = 0.004; right putamen, *p* = 0.001) (Table 2). Other SNPs (*rs11655548, rs9393,* and *rs821616*) had no genotype-by-diagnosis interaction. There was no significant gene-gene interaction on GM volume between *rs28365859* and *rs821616*.

**Table 2:** Effect of *rs28365859* genotype and genotype-by-diagnosis interaction on gray matter volume.

	Brain region	Contrast	Covariates	Talairach coordinate			Cluster size	*p*
				x	y	z		
Interaction on whole brain								
	Rt putamen		age, sex	32	13	−5	125	<0.0001 (uncorrected)
	Lt insula		age, sex	−39	10	−11	108	<0.0001 (uncorrected)
Interaction on SVC								
	Rt putamen		age, sex	32	13	−5	168	0.001 (FWE-corrected)
	Lt insula		age, sex	−39	10	−11	232	0.004 (FWE-corrected)
Genotype effect on SVC^a^								
	Rt putamen	ConC−>ConC+	age, sex	30	16	−1	60	0.023 (FWE-corrected)
	Lt insula	SzC+>SzC−	age, sex	−36	8	−11	52	0.047 (FWE-corrected)
		SzC+>SzC−	age, sex, doi,med	−36	8	−11	68	0.037 (FWE-corrected)

ConC+, controls with C allele; ConC−, controls without C allele; doi, duration of illness; FWE, family-wise error; Lt, left; med, daily medication dose; Rt, right; SVC, small volume correction; SzC+, schizophrenia patients with C allele; SzC−, schizophrenia without C allele.

a There were no suprathreshold clusters for other contrasts.

**Figure 2:**
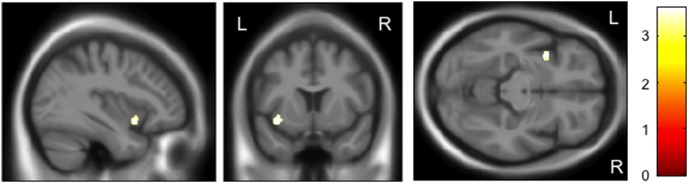
Impact of the *rs28365859* genotype on gray matter volume of left insula in schizophrenia. Age, sex, illness duration, and medication dose were used as covariates. The protective C allele carriers had a significantly larger left insula than the G homozygotes. Anatomical localizations are displayed on the normal template MR images in three directions. The color bar shows t values corresponding to the color in the figure.

**Figure 3:**
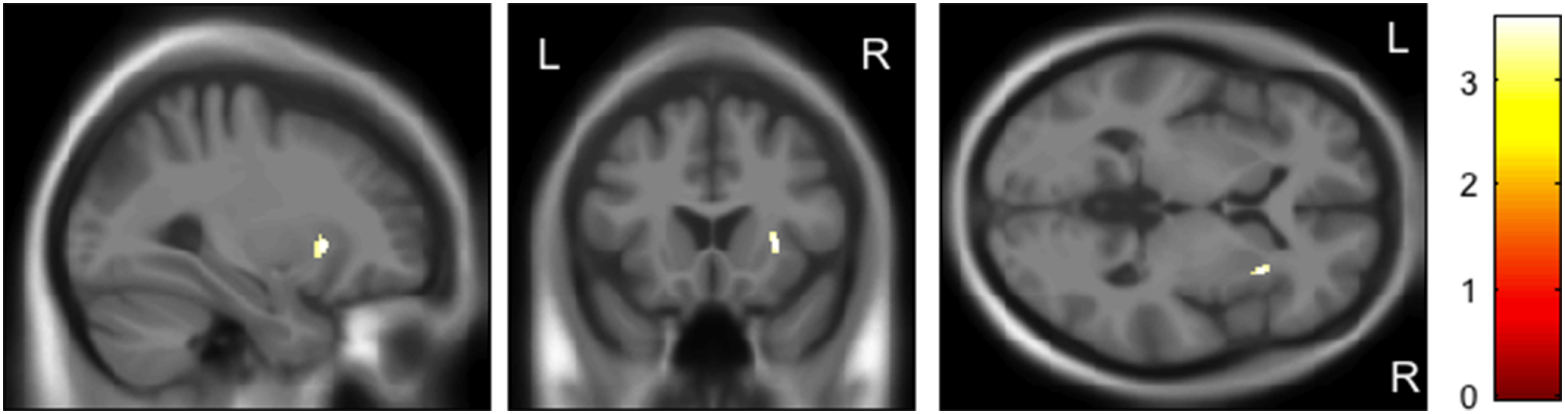
Impact of the *rs28365859* genotype on gray matter volume of the right putamen in healthy controls. The G allele homozygotes had a significantly larger right putamen than the C allele carriers. Anatomical localizations are displayed on the normal template MR images in three directions. The color bar shows t values corresponding to the color in the figure.

On the basis of significant genotype-by-diagnosis interactions of *rs28365859*, we then separately investigated its genotype effect on GM volume in schizophrenia and control groups. The protective C allele carriers had a significantly larger left insula than G homozygotes only for the schizophrenia patients (FWE-corrected *p* = 0.047, Fig. 2), while the controls with G allele homozygosity had a significantly larger right putamen than the C allele carriers (FWE-corrected *p* = 0.023, Fig. 3) (Table 2). The C allele was also related to smaller left insula in controls (FWE-corrected *p* = 0.144) and larger right putamen in schizophrenia patients (FWE-corrected *p* = 0.078), although these effects were not statistically significant. The findings reported herein did not change even when we added the illness duration and medication dose as covariates for the SVC analyses for the schizophrenia patients (Table 2).

### Hypothesis-driven ROI analysis for hippocampus

**Figure 4:**
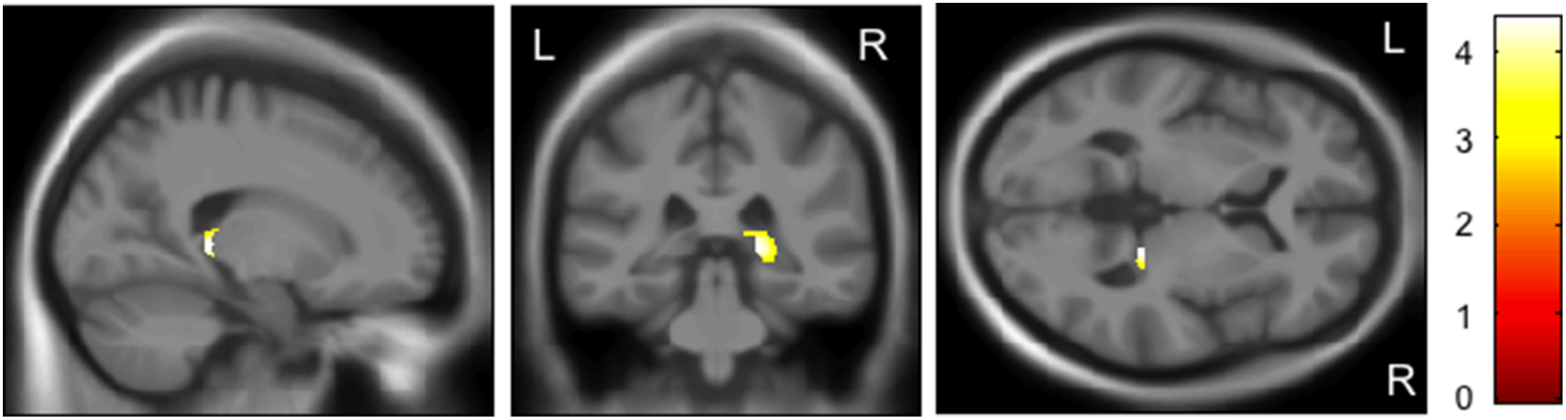
Impact of the *rs28365859* genotype on gray matter volume of the right hippocampus in schizophrenia. Age, sex, illness duration, and medication dose were used as covariates. The protective C allele carriers had a significantly larger right hippocampus than the G allele homozygotes. Anatomical localizations are displayed on the normal template MR images in three directions. The color bar shows t values corresponding to the color in the figure.

The protective C allele carriers of *rs28365859* had a significantly larger right, but not left, hippocampal volume than the G allele homozygotes (FWE-corrected *p* = 0.009, Table 3). For the analyses in each diagnostic group, such an effect of *YWHAE* genotype was significant only in schizophrenia patients (FWE-corrected *p* = 0.009, Table 3 and Fig. 4). That result in schizophrenia remained the same even when we added illness duration and medication as covariates (Table 3).

**Table 3:** Effect of *rs28365859* genotype on right hippocampal gray matter volume.

Contrast^a^	Covariates	Talairach coordinate			Cluster size	FWE *p*
		x	y	z		
C+>C−	age, sex	24	−35	0	120	0.009
						
SzC+>SzC−	age, sex	20	−33	3	78	0.009
	age, sex, doi, med	20	−33	3	120	0.002

C+, subjects with C allele; C−, subjects without C allele; doi, duration of illness; FWE, family-wise error; med, daily medication dose; SzC+, schizophrenia patients with C allele; SzC−, schizophrenia patients without C allele.

a There were no suprathreshold clusters for other contrasts.

## Discussion

This is the first structural MRI study to report the relationship between the functional polymorphism of *YWHAE*, a gene encoding 14-3-3epsilon, and brain morphology in patients with schizophrenia and healthy controls. While no significant difference was found in clinical and demographic data between the *YWHAE (rs28365859)* C allele carriers (protective allele group) and G allele homozygotes in both schizophrenia and control groups, the exploratory whole-brain analysis of regional GM volume demonstrated significant genotype-by-diagnosis interaction of *rs28365859* on the left insula and right putamen. Subsequent SVC analyses showed that the protective C allele carriers had a significantly larger left insula than G homozygotes only for the schizophrenia patients, while the controls with G allele homozygosity had a significantly larger right putamen than the C allele carriers. Furthermore, the hypothesis-driven ROI analysis revealed that the subjects with the C allele had a larger hippocampal volume, especially for schizophrenia patients. Our report using a Japanese cohort thus suggests that the genotype variation of 14-3-3epsilon, a *DISC1*-interacting molecule associated with neuronal development [13], [21], may be at least partly related to the abnormalities in brain morphology reported in schizophrenia. Importantly, we found no significant genotype effect of non-risk *YWHAE* SNPs (*rs11655548* and *rs9393*) on GM volume, supporting the specific role of *rs28365859* in the pathophysiology of schizophrenia [22].

Our finding of preserved insula GM volume in schizophrenia patients with protective C allele of *rs28365859* is consistent with the literature suggesting a significant role of insula pathology in schizophrenia [38]. GM reduction of the insula, which plays crucial roles in emotional and various cognitive functions as a component of the limbic integration cortex [39], has been repeatedly described in schizophrenia [40], [41]. GM reduction or dysfunction of the insula has also been implicated in the manifestation of psychotic symptoms and cognitive impairments [38]. The exact neurobiological basis for these GM changes of the insula in schizophrenia remains unknown, but the defects in gyrification [42], cytoarchitectural abnormalities [43], [44], and significant volume reduction prior to the illness onset [45], [46] imply early neurodevelopmental abnormalities in this region. A lack of insular GM abnormalities in non-psychotic co-twins within monozygotic twins discordant for schizophrenia [47] suggests that the insular findings in schizophrenia are also attributable to non-genetic factors. In this study, healthy controls with C allele had a non-significantly smaller left insula compared to G homozygotes. The reason for this opposite direction of volume changes related to the same allele between schizophrenia patients and controls is unclear, but our earlier MRI study demonstrated that the *DISC1 (rs821616)* genotype variation could also differently affect the insula GM volume in schizophrenia patients and healthy comparisons [18]. The current evidence for *DISC1* alone as a genetic risk factor of schizophrenia is not strong [20]. Indeed, the present study did not support its effect on brain morphology in schizophrenia. However, considering that *DISC1* interacts with a complex formed by related molecules (including 14-3-3epsilon) during processes involved in neuronal development, such as axonal elongation [13], the present results raise the possibility that the genetic variation of *DISC1*-interacting molecules might have an additive or independent role in alterations of the neural development in schizophrenia, especially regarding the insula pathology [38]. The potential role of genetic variation in *DISC1*-interacting molecules and its interaction with other genetic/non-genetic factors in the pathophysiology of schizophrenia should be further tested through *in vitro* and *in vivo* studies.

We also found significant *rs28365859* genotype-by-diagnosis interaction on the right putamen, with the C allele carriers having a smaller putamen volume only for healthy subjects. This finding might have some association with a previous MRI study that demonstrated the relationship between functional *DISC1* genotype and striatal volume [48]. Taken together with animal data that the *DISC1* gene influences striatal dopamine receptor levels [49], Chakravarty et al. [48] hypothesized that a key risk pathway for schizophrenia might be conferred via *DISC1*’s effects on the striatum. MRI findings of the putamen in schizophrenia have been highly controversial; smaller [50] or normal [51], [52] volume was reported in first-episode antipsychotic-naïve patients, with both volume expansion [51], [53] and decrease [54] following antipsychotic treatment. We did not find a significant effect of the genetic variation of 14-3-3epsilon, a *DISC1*-interacting molecule, on the basal ganglia in our sample of chronically medicated schizophrenia patients. However, the possible role of genetic variation of *DISC1* and its interacting molecules on brain morphology in schizophrenia should be examined in future, ideally using a larger antipsychotic-naïve sample.

In this study, as hypothesized, we also demonstrated that the subjects with the protective C allele of r*s28365859* had a larger hippocampal volume, especially for schizophrenia patients. Hippocampal GM volume is thought to represent an endophenotype associated with the clinical expression of schizophrenia [55]. Brain imaging studies suggest that variants in the *DISC1* gene may influence normal neurodevelopment, brain structure, function, and neurochemistry, but the association of the common *DISC1* SNPs with hippocampal regions has been inconsistent for both schizophrenia and healthy subjects (reviewed by Duff et al. [19]). However, the expression of *DISC1*-binding partners such as *NUDEL* and *LIS1*, which form a complex with 14-3-3epsilon [13], [21], is reduced in the hippocampus of postmortem schizophrenia brains [56]. More specifically, animal studies using genetically modified 14-3-3epsilon-deficient mice showed developmental defects of hippocampal neurons [21] as well as behavioral changes related to clinical features of schizophrenia (i.e., anxiety-like behavior, working memory deficits) [22]. Schizophrenia is a complex disorder with a variety of pathologies and risk factor genes, and the variation of a single gene could explain only a part of its clinical expression. We found no direct interaction between the *YWHAE* (*rs28365859*) and *DISC1* (*rs821616*) SNPs on gray matter volume in schizophrenia in this study. Nevertheless, the present and previous basic studies suggest the possibility that genetically defined impairment of *DISC1* and/or 14-3-3epsilon could cause neuronal developmental defects in brain regions including the hippocampus, which result in the increased risk of developing schizophrenia.

There are several confounding factors in the present study. First, in contrast to recent large multinational consortium genome-wide association studies [57], [58], this study examined the effect of the *YWHAE* genotype only in a relatively small Japanese sample. Our whole-brain analysis found a specific *YWHAE* genotype effect only on the left insula in schizophrenia, but the current study was potentially underpowered to detect significant genotype effects on other brain regions owing to the small sample size. For example, the relation between the protective C allele of *rs28365859* and larger hippocampal volume in all subjects (but more robust in schizophrenia patients) was detectable only by the hypothesis-driven ROI analysis, which is thought to be more sensitive than whole-brain analysis. Furthermore, an animal study by Sekiguchi et al. [59] suggested a relationship between the defect of 14-3-3epsilon and axon elongation abnormality in the prefrontal cortex. As we also found mild diagnosis-by-genotype interaction in frontal regions when we used a significance level of uncorrected *p*<0.001 in exploratory whole-brain analysis (data not shown), future studies on a larger sample of schizophrenia might detect other *YWHAE* genotype effects on brain morphology including the frontal regions. Second, we examined schizophrenia patients with an illness duration of approximately 5 years in this study. Illness chronicity [60] and medication with antipsychotics [61], [62] could significantly affect brain morphology. Although there was no difference in these variables between the patients with and without the C allele of *rs28365859* (Table 1) and we statistically controlled these factors, the present findings should be replicated using patients at early illness stages. Third, the current study cannot address the disease specificity of our *YWHAE* findings. There are overlapping GM structural abnormalities in the neurobiology of schizophrenia and bipolar disorder [63] and there are several susceptibility genes (e.g., *DISC1*) for both of these disorders [19]. Finally, considering that we examined only four selected SNPs in the present study, more comprehensive assessment would be required to clarify the role of genetic variation of *DISC1* and its interacting molecules in the pathophysiology of schizophrenia.

In conclusion, we found that the C allele of *YWHAE* (*rs28365859*) is related to preserved GM volume of the insula and hippocampus in schizophrenia, major brain regions related to the illness, in a Japanese sample. These findings are likely to provide neurobiological support for previous genetic and expression studies suggesting that this SNP reduces the risk of schizophrenia [22].

## Supporting Information

Figure S1 **Diagnosis effect on gray matter volume in all subjects analyzed by using the SPM8 full factorial model.** Age and sex were used as covariates. Healthy controls had a larger gray matter volume compared with schizophrenia patients predominantly in fronto-temporo-limbic regions (family-wise error-corrected *p*<0.05). Anatomical localizations are displayed on the normal template MR images in three directions. The color bar shows t values corresponding to the color in the figure.
(TIFF)

Table S1 **Diagnosis effect on gray matter volume in all subjects.** Each region was defined using the Automated Anatomical Atlas (AAL) atlas [33].
(DOCX)
