# Supplementary figures and images for: The Polymorphism of YWHAE, a Gene Encoding 14-3-3Epsilon, and Brain Morphology in Schizophrenia: A Voxel-Based Morphometric Study
Source: PLoS One. 2014 Aug 8;9(8):e103571. doi: 10.1371/journal.pone.0103571 (PMC4126687; doi:10.1371/journal.pone.0103571)

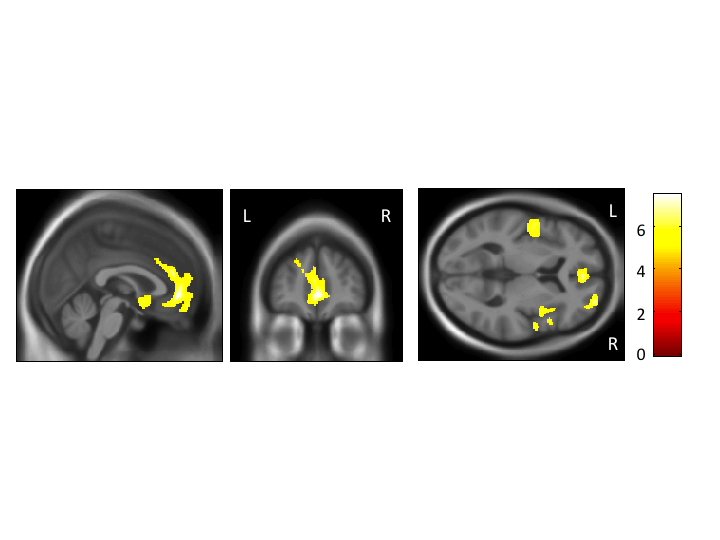

Supplement: Figure S1 — Diagnosis effect on gray matter volume in all subjects analyzed by using the SPM8 full factorial model. Age and sex were used as covariates. Healthy controls had a larger gray matter volume compared with schizophrenia patients predominantly in fronto-temporo-limbic regions (family-wise error-corrected p<0.05). Anatomical localizations are displayed on the normal template MR images in three directions. The color bar shows t values corresponding to the color in the figure. (TIFF) [file pone.0103571.s001.tiff]
