# Supplementary material for: The Polymorphism of YWHAE, a Gene Encoding 14-3-3Epsilon, and Brain Morphology in Schizophrenia: A Voxel-Based Morphometric Study
Source: PLoS One. 2014 Aug 8;9(8):e103571. doi: 10.1371/journal.pone.0103571 (PMC4126687; doi:10.1371/journal.pone.0103571)
Supplement: Supplementary file 2 [file pone.0103571.s002.docx]

Table S1

Diagnosis effect on gray matter volume in all subjects.

Brain region[a)]	Talairach coordinates			Cluster size	FWE-corrected *p*
	x	y	z		
Lt superior frontal gyrus	-11	59	14	3970	<0.001
Rt insula	42	11	-1	2256	<0.001
Rt superior frontal gyrus	29	58	-2	374	<0.001
Lt superior temporal gyrus	-53	-3	1	551	<0.001
Rt precuneus	11	-52	26	39	0.005
Lt insula	-42	14	-5	102	0.005
Rt cingulate gyrus	12	5	34	54	0.013
Lt inferior frontal gyrus	-52	10	17	22	0.014
Lt cingulate gyrus	-6	-31	38	23	0.016
Rt postcentral gyrus	61	-16	23	21	0.024
Lt anterior cingulate gyrus	-5	20	-3	5	0.033
Lt middle frontal gyrus	-27	53	8	8	0.034
Rt superior frontal gyrus	27	48	25	6	0.040
Rt middle temporal gyrus	65	-30	-10	2	0.045

FWE, family-wise error; Lt, left; Rt, right.

[a)] Larger in controls than in schizophrenia patients. There was no cluster showing a larger gray matter volume in schizophrenia patients than in controls.
